# Reliability and validity of the Chinese Version of the Frequency, Intensity, and Burden of Side Effects Rating

**DOI:** 10.3389/fpsyt.2025.1613331

**Published:** 2025-09-24

**Authors:** Jie Zhao, Junyu Ji, Aiai Cao, Jing Ni, Tongdan Cao

**Affiliations:** ^1^ Department of Psychiatry, Huangpu District Mental Health Center, Shanghai, China; ^2^ Department of Rehabilitation, Chongming Hospital Affiliated to Shanghai University of Medicine and Health Sciences, Shanghai, China; ^3^ The Department of Psychiatry, The Third People's Hospital of Foshan, Foshan, Guangdong, China

**Keywords:** depressive disorder, side effects, frequency, intensity, burden, rating scales

## Abstract

**Background:**

The Frequency, Intensity, and Burden of Side Effects Rating (FIBSER) Scale is a brief tool developed to assess side effects in patients with major depressive disorder (MDD). This study aims to evaluate the reliability and validity of the Chinese version of the FIBSER in Chinese patients with MDD.

**Methods:**

A total of 162 MDD patients were enrolled to assess the Chinese version of FIBSER, the self-report version of the Quick Inventory of Depressive Symptoms (QIDS-SR16), and the Rating Scale for Side Effects (SERS). Reliability and validity were evaluated using Cronbach’s α coefficient, test-retest reliability, Spearman correlation analysis, and Principal component analysis.

**Results:**

The Cronbach’s α coefficient for the Chinese version of the FIBSER was 0.945. The test-retest reliability coefficients for the three domains were 0.799, 0.821, and 0.897, respectively. The correlation coefficients between FIBSER and SERS ranged from 0.765 to 0.817. The correlation coefficients with QIDS-SR16 ranged from 0.187 to 0.270. Principal component analysis found one significant principal component, highlighting the prominence of side effect frequency as a factor. The Composite Reliability and Average Variance Extracted values in the confirmatory factor analysis showed excellent convergent validity.

**Conclusions:**

The study suggests that the Chinese version of the FIBSER tool exhibits good reliability and validity in patients with MDD. It is suitable for the rapid clinical assessment of side effects in patients receiving antidepressant treatment.

## Introduction

Major depressive disorder (MDD)is widely recognized as common, impairing social functioning and economic productivity, and diminishing quality of life (1–3). According to the Global Burden of Disease survey, depressive disorders were major contributors to years lived with disability (YLDs), ranking second in 2019 (4, 5). The World Health Organization predicts that depression will become the leading cause of global disease burden by 2030 (6).

Given the severity of the epidemiological burden, antidepressant treatment becomes critically important to reduce recurrence risk. An antidepressant medication is recommended as an important treatment choice for patients with mild to moderate major depressive disorder. It is well known that side effects commonly occur during antidepressant treatment (7–9)and are reported to be one of the main reasons for discontinuing medication (10). Clinicians inform patients about potential side effects before prescribing and monitor the side effects during both the acute phase and maintenance phase (11, 12). The side effect profiles vary among different classes of antidepressants (13, 14). Gastrointestinal and sexual symptoms are frequently reported in first-line antidepressants, including gastrointestinal dysfunction, sexual dysfunction, somatic anxiety, and sleep disturbances (13, 15, 16).

The Frequency, Intensity, and Burden of Side Effects Rating (FIBSER) is free and simple, designed to be concise and applicable to antidepressant users of different ages and genders. The scale is flexible, enabling patients to quickly and accurately describe their drug response. The content of the scale is simple and easy to understand, making it convenient for patients to complete the self-assessment (17–19). FIBSER was developed to assess antidepressant treatment side effects in the Sequenced Treatment Alternatives to Relieve Depression (STAR*D) project (20). In the Canadian Network for Mood and Anxiety Treatments (CANMAT), FIBSER (20), UKU Side Effect Rating Scale (14), and Toronto Side Effects Scale (21) are recommended to monitor side effects (12). The FIBSER indicates good reliability and validity among foreign populations (Cronbach’s α 0.93) (20). Through the self-assessment, doctors can more quickly understand drug responses, thereby more effectively adjusting the treatment plans. This helps to reduce the consultation time and improve clinic efficiency. The use of self-rating scales allows patients to be more actively involved in their treatment process. Some studies have shown that self-rated scales are more sensitive than clinician-rated scales in assessing the influence of antidepressant medication side effects on patients (21, 22). The scale helps both parties to understand the needs and expectations more clearly.

In Chinese culture, there is a prevalent concern among patients regarding the potential adverse effects of medications, especially in the field of psychiatry. Patients may fear side effects due to a lack of understanding or misinformation. To address this, it is crucial to enhance patient education and communication. Providing detailed information about how side effects can be managed can improve their willingness to adhere to treatment. However, due to the large outpatient volume, the time allocated to each patient by physicians to assess side effects is restricted. In regions with limited medical resources, there is a pressing need for simple and effective tools to monitor side effects in patients. Currently, there is no self-rating scale available for rapid assessment of side effects in China. Interestingly, when treating depression with physical therapy such as acupuncture in traditional Chinese medicine, no scales adequately meet the needs of monitoring adverse reactions to antidepressant treatment (23–25).

Therefore, this study aims to explore the reliability and validity of FIBSER in Chinese patients with Major Depressive Disorder.

## Methods

### Study design

Participants were recruited from both inpatients and outpatients at the Huangpu District Mental Health Center in Shanghai and the Third People’s Hospital of Foshan, between January 2021 and December 2023. A total of 162 individuals were included. Patients meeting DSM-5 criteria for MDD, with a current major depressive episode (MDE), and taking antidepressant medication were eligible to be invited to participate in the study. Before conducting this study, the research protocol and procedures had been approved by the Ethics Committee of the Huangpu District Mental Health Center. They had followed the relevant provisions of the Declaration of Helsinki. A psychiatrist explained the procedures in lay language to the participants to give them a full sense of the research protocol and procedures. Written informed consent was obtained from all participants. In the face-to-face interview, trained psychiatrists recorded the basic sociodemographic and clinical symptoms questionnaire.

### Participant inclusion and exclusion criteria

Inclusion criteria:1) aged≥18 years old; 2) met the Diagnostic and Statistical Manual of Mental Disorders, (DSM-5 fifth edition) diagnosis of major depressive disorder by a psychiatrist.;3)taking antidepressant medication at least two weeks; Due to the reliability and safety of the assessment and ethical reasons, exclusion criteria included (1) lifetime history of bipolar disorder or psychotic disorder; (2) physical therapy for depression within one month before the study; (3) females in pregnancy or breast-feeding period. (4) Unable to read and understand patient self-assessment questionnaires;

### Measurements

Baseline measures collected by the psychiatrist included standard sociodemographic information and the type and duration of antidepressant use.

FIBSER was a self-report assessing three domains of medication side effect impact: Frequency, Intensity, and Burden (in **Supplementary Material 1**). After being authorized by Stephen R. Wisniewski, the development of the Chinese version of FIBSER began with translation from English to Chinese by two psychiatrists proficient in both languages (in **Supplementary Material 2**). This initial translation was followed by a back-translation into English by a professional proficient in English (in **Supplementary Material 3**). Subsequently, it was confirmed by the two psychiatrists to ensure fidelity. This process culminated in the finalization of the Chinese version of FIBSER.

The severity of depression was evaluated by the self-report version of the Quick Inventory of Depressive Symptoms (QIDS-SR16) (26). The side effects were assessed using the SERS (27, 28). These assessment questionnaires provided comparative data used in assessing the psychometric characteristics of the FIBSER.

After patients are enrolled, sociodemographic characteristics and scale assessments are conducted. According to the random sequence table prepared by the third party, a random sample of 32 patients underwent retesting using the Chinese version of FIBSER after 2 weeks to evaluate test-retest reliability. The data collectors were blinded to previous scores to avoid bias. The study had a small sample size, and there was no missing data.

### Statistical analysis

All analyses were conducted using the Statistical Package for Social Sciences (SPSS) version 25.0. Internal consistency of the FIBSER was assessed using Cronbach’s α coefficient. 32 patients were randomly sampled to retest FIBSER to verify the reliability of the FIBSER retest. Validity was further assessed using Principal Component Analysis and Confirmatory Factor Analysis. Spearman correlation coefficients were calculated between the FIBSER, SERS, and QIDS-SR16. All p-values were two-tailed, with significance was defined as p < 0.05.

## Results

### Demographic and clinical characteristics of participants

A total of 162 participants were included in the analysis, among whom 115 (71%) were female. The mean age was 32.48 years (SD = 16.45), and the mean age of onset in years was 28.75 years (SD = 14.47). The mean duration of antidepressant use was 6.71 months (SD = 20.53). The QIDS-SR16 total score was 14.41 ± 5.81, and the SERS total score was 3.78 ± 4.10. The socio-demographic characteristics and clinical features of participants were summarized in **Table 1**.

**Table 1:** Sociodemographic characteristics and clinical features of participants.

Characteristics	n (%)	Mean ± SD	Range
Sociodemographic characteristics			
Age (years)		32.48 ± 16.45	18-76
Sex			
Male	47(29)		
Female	115(71)		
Education (years)		13.88 ± 3.29	2-22
Marital status			
Single	90(55.6)		
Married/cohabiting	72(44.4)		
Occupational status			
Unemployed	40(24.7)		
Employed or student	122(75.3)		
Smoking status			
Never smoker	147(90.7)		
Current smoker	15(9.3)		
Family history	16(9.9)		
Drinking status			
Never drinker	149(92.0)		
Current drinker	13(8.0)		
Somatic comorbidity	30(18.5)		
Age of onset in years		28.75 ± 14.47	10-70
Duration of illness (months)		36.85 ± 59.27	0.5-480
Duration of antidepressant use (months)		6.71 ± 20.53	0.5-120
Scales scores			
QIDS-SR16 total score		14.41 ± 5.81	2-25
SERS total score		3.78 ± 4.10	0-18

Among the participants, 45.7% of participants used escitalopram (10–20 mg/day). Venlafaxine (75–225 mg/day) use was at 38.9%, while duloxetine (40–60 mg/day) was reported at 4.9%. Paroxetine (20–40 mg/day) and sertraline (50-200mg/day) were used by 3.7% each. Mirtazapine (15–30 mg/day) use was at1.2%. Additionally, trazodone(100mg/day), fluvoxamine(100mg/day), and Fluoxetine(20mg/day) were each reported at 0.6%.

### The internal consistency of the FIBSER

The distribution of the FIBSER scores across the frequency, intensity, and burden domains is reported in **Table 2**.

**Table 2:** Distribution of FIBSER scores for each item.

Frequency	No side effects	Present 10% of the time	Present 25% of the time	Present 50% of the time	Present 75% of the time	Present 90% of the time	Present all the time
n (%)	45(27.8)	58(35.8)	37(22.8)	13(8.0)	4(2.5)	2(1.2)	3(1.9)
Intensity	No side effects	Trivial	Mild	Moderate	Marked	Severe	Intolerable
n (%)	44(27.2)	54(33.3)	39(24.1)	17(10.5)	6(3.7)	2(1.2)	0(0)
Burden	No impairment	Minimal impairment	Mild impairment	Moderate impairment	Marked impairment	Severe impairment	Unable to function
n (%)	65(40.1)	51(31.5)	22(13.6)	13(8.0)	6(3.7)	4(2.5)	1(0.6)

The Cronbach’s α coefficient for the Chinese version of FIBSER was 0.945, indicating excellent internal consistency.

### The test-retest reliability of the FIBSER

32 participants were reassessed by the FIBSER two weeks later, and the test-retest reliability was analyzed by Spearman correlation analysis. The results showed that the test-retest reliabilities of the Chinese version of FIBSER in three domains of medication side effect impact (Frequency, Intensity, and Burden) were 0.799, 0.821, and 0.897, respectively, all p < 0.01. The Cohen’s d values indicated small effect sizes across the FIBSER dimensions, with values of 0.492 (Frequency), 0.484 (Intensity), and 0.473 (Burden).

### The validity of the FIBSER

As shown in **Table 3**, the Spearman correlation between FIBSER and SERS ranged from 0.765 to 0.817. The Spearman correlation between FIBSER and QIDS-SR16 ranged from 0.187 to 0.270. The Chinese version of FIBSER was highly correlated with the SERS. Conversely, FIBSER shows a lower correlation with QIDS-SR16 (all p < 0.05).

**Table 3:** Correlation of FIBSER with the SERS and QIDS-SR16.

	Correlation	
FIBSER	SERS	QIDS-SR16
Frequency	0.817	0.270
Intensity	0.803	0.261
Burden	0.765	0.187

SERS, Rating Scale for side effects.

QIDS-SR_16_, 16-item, Quick Inventory of Depressive Symptoms-Clinician Administered version.

### Principal component analysis and confirmatory factor analysis

The research indicated that a linear correlation existed among the three research variables (the correlation coefficients were 0.828, 0.861, and 0.878, respectively), and the data structure was reasonable. The KMO test coefficient was 0.766, with individual item KMO test coefficients were 0.775, 0.721, and 0.809, all greater than 0.7. The Bartlett test result was P < 0.001. These results showed the suitability of the data for principal component analysis.

**Figure 1:**
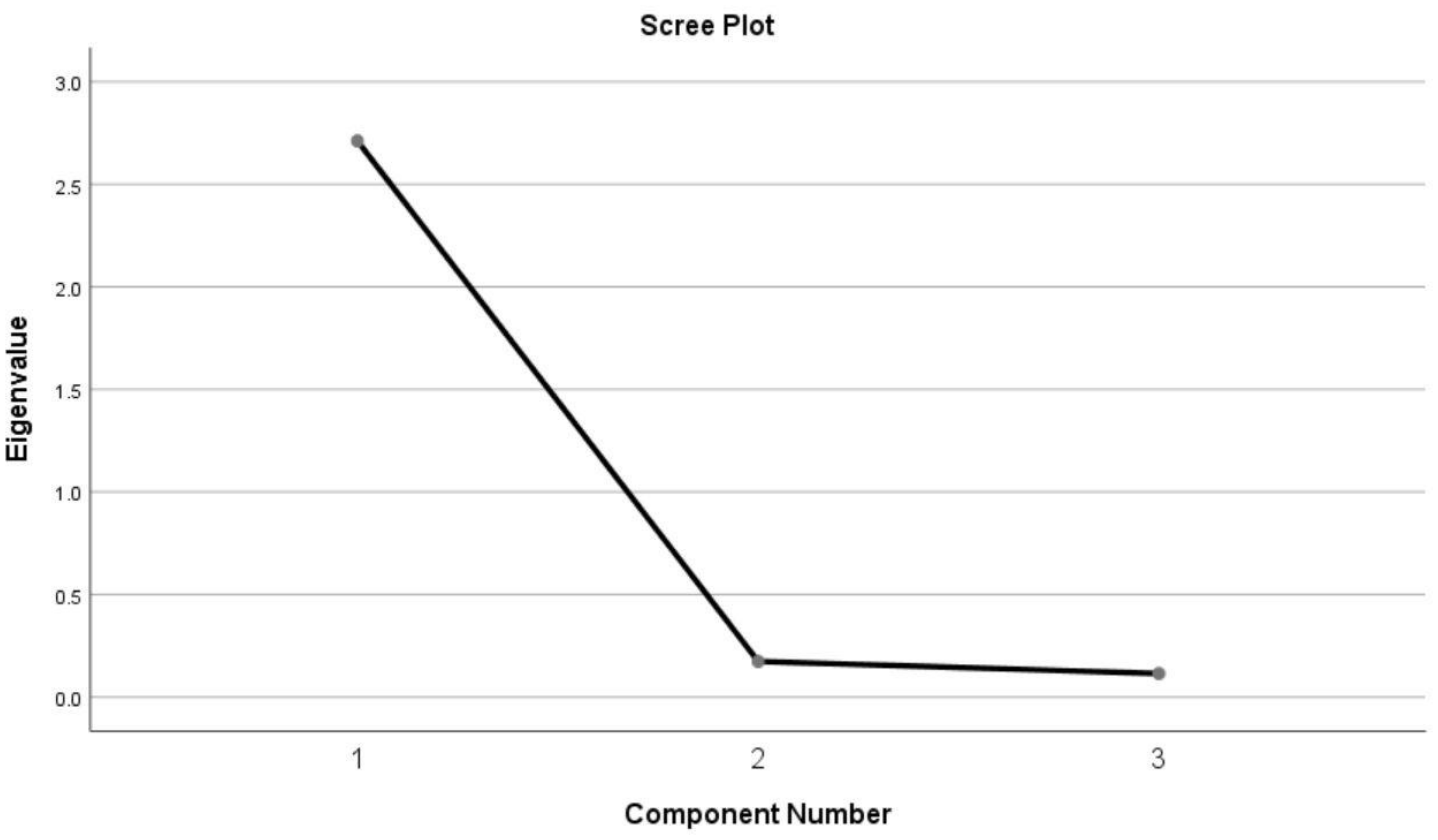
Scree plot from principal components analysis of FIBSER.

The results of principal component analysis showed one major component with an eigenvalue greater than 1(the value was 2.711), accounting for 90.376% of the total variance (**Figure 1**). The FIBSER scale could potentially be shortened to focus on the side effect frequency item. The scree plot could provide a relatively comprehensive explanation.

We also conducted a confirmatory factor analysis (CFA) using Amos28. A one-factor model was specified, with the three items loading onto a single latent factor. The CFA provides crucial information on the model’s parameters: All standardized factor loadings were high: Frequency Item (λ = 0.919), Intensity Item(λ = 0.956), Burden Item (λ = 0.901). We calculated the Composite Reliability (CR = 0.947) and Average Variance Extracted (AVE = 0.857) based on the CFA estimates. Both values demonstrated excellent convergent validity.

## Discussion

The FIBSER is considered one of the most convenient tools for side effects. The findings of this study suggested that the Chinese version of the FIBSER shows good reliability and validity, supporting its use as a rapid screening tool for antidepressant side effects.

In this study, the Cronbach’s alpha coefficient for the Chinese version of FIBSER was 0.945, indicating excellent internal consistency. The results of this study indicated that the Chinese version of the FIBSER exhibited good test-retest reliability, suggesting the stability of its short-term assessment outcomes. A previous report showed that the Cronbach α of the FIBSER ranged from 0.91 to 0.93 (20). The Cronbach’s α values were consistently greater than 0.9, indicating some redundancies among the items. The Composite Reliability and Average Variance Extracted values in the confirmatory factor analysis showed excellent convergent validity. The three-item structure of the FIBSER scale was relatively brief (3 items for 1 factor, resulting in degrees of freedom was 0), which made it difficult to calculate the statistical values for the standard model fit in the structural equation model analysis.

The Principal Component Analysis result showed that “frequency” accounted for 90% of the variance, which was in line with our expectations, further supporting the assumption that the scale items may be redundant. In our study, the “frequency” variable is the main driver of data variation, which might be related to the characteristics of the sample. Although the authors of the FIBSER scale did not use Principal Component Analysis, they also considered shortening the instrument to focus on the side effect burden question.

In the FIBSER scale scoring, the frequency and intensity items are excluded from the final scoring; only the burden item is included. Scores of 0–2 indicate that continue the current treatment unless there is a problem with safety or symptom control. Scores of 3–4 suggest the side effects require further treatment through interventions such as dose reduction. Scores of 5–6 indicate that a change in treatment plan, including dose reduction or changing medications.

Although the full scale contains some redundancy, it includes only three questions, each of which addresses patient concerns. Our study described the distribution of FIBSER scores for each item, suggesting that some side effects were highly frequent but only of modest intensity and burden to the patient. We found one more person with no side effects in frequency than in intensity (45 VS 44). In the STAR*D study, over 14 weeks of follow-up, more participants had no side effects in the frequency domains than in the burden domains at each observation (31.0% VS 30.6%; 36.2% VS 35.6%). It indicated that some side effects may occur infrequently, but the patient still felt unwell (20). Escitalopram, venlafaxine, and duloxetine were the three most used antidepressant drugs in this study (accounting for 89.5%). Doctors preferred to prescribe them due to their greater safety and tolerability (29–31). Selective serotonin reuptake inhibitors commonly cause nausea and vomiting (12), which tend to dissipate over the first few weeks of treatment. If using the single-item version, whether it is the frequency or the burden items, some important information will be missed. The full scale provides clinicians with information to monitor side effects and refine the treatment plan for individual patients.

In terms of validity, our study found that the Chinese version of the FIBSER suggested good validity. FIBSER showed a high correlation with the SERS, indicating good criterion validity and its ability to assess the side effects of antidepressant medications effectively. In a previous study, correlations between the PRISE and the FIBSER ranged from 0.22 to 0.32 (20). Although the SERS was initially developed for tricyclic antidepressants and includes 14 domains, with one domain optionally self-administered, this might explain their high correlation. Our study also found that the Chinese version of the FIBSER had a lower correlation with the QIDS-SR16 symptom score, suggesting that FIBSER scores could effectively differentiate side effects from depression symptom ratings. These findings are consistent with previous research on the validity of the FIBSER (20).

A total of 162 participants were included in the study, with a female predominance (71%). It might be associated with the gender distribution among patients with depression disorder. Previous studies showed that female patients had a higher prevalence of any depressive disorders (32)and were more likely to seek treatment in clinical settings (33, 34). The gender imbalance might limit the generalizability of the findings to male participants. Future studies might aim to recruit a more balanced gender ratio.

The shortcoming of FIBSER is that specific side effects are not identified. In clinical practice, if a moderate burden is identified, it will be brought to the attention of the clinician, and some intervention will be considered.

## Limitations

Some limitations should be mentioned. The dimension scores of patients in the study focused on mild to moderate levels, and the frequency, intensity, and burden of severe side effects were relatively low.

The FIBSER scale consisted of only three items, which inherently restricted the complexity of the factor model that could be tested. The sample size was relatively modest, and the gender imbalance might limit the generalizability of the findings. For the assessment of depressive symptoms, we only used self-rating scales, lacking clinician-rated measures. The cross-sectional design also acted as a limiting factor for establishing sensitivity to change. Although translation and back-translation ensured linguistic accuracy, the lack of quantitative measures of content validity (such as content validity index or expert ratings) limited a comprehensive evaluation of the items.

## Conclusion

In summary, the results of this study suggest that the Chinese version of FIBSER describes good reliability and validity as a self-report measure of side effects in patients receiving treatment for depression. The Chinese version of the FIBSER is easy to operate, concise, and suitable for rapid clinical assessment.

## Data Availability

The raw data supporting the conclusion of this article will not be available to protect participant confidentiality and privacy. Requests to access the datasets should be directed to the corresponding authors.
