# Supplementary material for: Reliability and validity of the Chinese Version of the Frequency, Intensity, and Burden of Side Effects Rating
Source: Front Psychiatry. 2025 Sep 24;16:1613331. doi: 10.3389/fpsyt.2025.1613331 (PMC12506080; doi:10.3389/fpsyt.2025.1613331)
Supplement: Supplementary file 1 [file DataSheet1.pdf]

Frequency, Intensity, and Burden of Side Effects Ratings (FIBSER)

Instructions: **Select the best response for the following three questions.**

1. Choose the response that best describes the frequency (how often) of the side effects of the medication you have taken within the past week for your depression. Do not rate side effects if you believe they are due to treatments that you are taking for medical conditions other than depression. Rate the frequency of these side effects for the past week.

No side effects	***Present 10% of the time***	***Present 25% of the time***	***Present 50% of the time***	***Present 75% of the time***	***Present 90% of the time***	***Present all of the time***
☐	☐	☐	☐	☐	☐	☐
0	1	2	3	4	5	6

2. Choose the response that best describes the intensity (how severe) of the side effects that you believe are due to the medication you have taken within the last week for your depression. Rate the intensity of the side effect(s), when they occurred, over the last week.

No side effects	***Trivial***	***Mild***	***Moderate***	***Marked***	***Severe***	***Intolerable***
☐	☐	☐	☐	☐	☐	☐
0	1	2	3	4	5	6

3. Choose the response that best describes the degree to which antidepressant medication side effects that you have had over the last week have interfered with your day-to-day functions.

No impairment	***Minimal impairment***	***Mild impairment***	***Moderate impairment***	***Marked impairment***	***Severe impairment***	***Unable to function***
☐	☐	☐	☐	☐	☐	☐
0	1	2	3	4	5	6

FIBSER in Clinical Practice

The response to question 3 may suggest that a change in treatment is necessary.
Q3 Score 0-2: Treatment should continue unless concerns exist about safety or symptom severity.
Q3 Score 3-4: The side effect should be addressed (eg, decrease dose).
Q3 Score 5-6: Change treatment (eg, decrease dose or switch medication).
Trivedi MH. *J Clin Psychiatry*. 2009;70 Suppl 6:26-31.

The FIBSER was used in the STAR*D trials in conjunction with the PRISE (Patient-Rated Inventory of Side Effects).
The FIBSER is in the public domain. It may be copied and used without permission.
Source: Wisniewski SR, et al. *J Psychiatr Pract*. 2006;12:71-79.
