# Supplementary material for: Reliability and validity of the Chinese Version of the Frequency, Intensity, and Burden of Side Effects Rating
Source: Front Psychiatry. 2025 Sep 24;16:1613331. doi: 10.3389/fpsyt.2025.1613331 (PMC12506080; doi:10.3389/fpsyt.2025.1613331)
Supplement: Supplementary file 2 [file Table1.docx]

**抗抑郁药物副反应频率、强度和负担量表**

**指导语：请务必仔细阅读以下内容**

请仔细回想你**过去一周内您由于抑郁而服用的药物**是否存在副反应，并评估相应的频率及程度。如果你感受到的副反应由其他疾病的治疗引起，本量表暂不予以考虑。**请在相应方框“□”内打“√”**

完成本量表约耗时3分钟，将有助于医生全面了解你的病情，请按照你的真实感受，独立填写。

所有问题的选择没有好、坏或对、错之分，你无需顾虑。如果你对某个问题无法确定，请选择最为接近的答案，如有任何问题请咨询你的医生。

1、请描述在**最近一周内，****您由于****抑郁而服用的药物**产生的副反应频率（出现副反应的时间占总时间的百分比）。**不包括您认为是因抑郁之外的疾病正在治疗而产生的副反应**。请圈出最符合你最近一周感受的副反应频率。

| 无副反应 | 占10%时间 | 占25%时间 | 占50%时间 | 占75%时间 | 占90%时间 | 始终出现 |
| --- | --- | --- | --- | --- | --- | --- |
| 🞏 | 🞏 | 🞏 | 🞏 | 🞏 | 🞏 | 🞏 |
| 0 | 1 | 2 | 3 | 4 | 5 | 6 |

2、请描述在**最近一周内，您认为是****由于抑郁而服用的药物**而产生的副反应的严重程度。请圈出最符合你最近一周感受的副反应严重程度。

| 无副反应 | 极微 | 轻度 | 中度 | 显著 | 严重 | 无法忍受 |
| --- | --- | --- | --- | --- | --- | --- |
| 🞏 | 🞏 | 🞏 | 🞏 | 🞏 | 🞏 | 🞏 |
| 0 | 1 | 2 | 3 | 4 | 5 | 6 |

3、请描述在**最近一周内，您由于抑郁而服用药物而产生的副反应对**日常生活功能的影响程度。请圈出最符合你最近一周感受的影响程度。

| 无影响 | 极微影响 | 轻度影响 | 中度影响 | 显著影响 | 严重影响 | 日常生活  无法进行 |
| --- | --- | --- | --- | --- | --- | --- |
| 🞏 | 🞏 | 🞏 | 🞏 | 🞏 | 🞏 | 🞏 |
| 0 | 1 | 2 | 3 | 4 | 5 | 6 |

**评分标准**

问题1和问题2（频率和强度）可为临床医生提供信息，但**它们不用于最终评分**

最终评分仅来自于问题3——负担，分值的临床意义如下：

0-2 =可继续当前治疗，除非存在安全性或症状控制的问题

3-4 = 副反应需要进一步处理（如减少剂量）

5-6 = 更改治疗方案（如减少剂量或换药）
