# Supplementary material for: Reliability and validity of the Chinese Version of the Frequency, Intensity, and Burden of Side Effects Rating
Source: Front Psychiatry. 2025 Sep 24;16:1613331. doi: 10.3389/fpsyt.2025.1613331 (PMC12506080; doi:10.3389/fpsyt.2025.1613331)
Supplement: Supplementary file 3 [file Table2.docx]

**Scale for frequency, severity and burden of antidepressant side effects**

**Directions: Please be sure to read the content below carefully**

Please carefully recall whether your **medication for depression in the past week** has side-effect and assess its frequency and degree. If the side-effects you feel are caused by the treatment of other diseases, this scale will not be considered for the time being. **Please tick "√" in the corresponding box "□".**

This form will take 3 minutes to complete and will assist doctors to fully understand your condition, please complete this honestly and independently.

For all the answers for the questions, there are no good or bad, or correct or incorrect; you need not worry. If you cannot confirm a particular question, please choose the closest answer. Please consult your doctor if you have any questions.

1. Please describe the frequency of side-effects (the percentage of time when side-effects occur as a percentage of total time) **for medications you take due to depression** **during the last week**. **Do not include side-effects that you believe are due to treatment of a disease other than depression.** Please circle the frequency of side-effects that best match your past week's experience.

| No side effects | 10% of the time | 25% of the time | 50 of the time | 75% of the time | 90% of the time | Always |
| --- | --- | --- | --- | --- | --- | --- |
| 🞏 | 🞏 | 🞏 | 🞏 | 🞏 | 🞏 | 🞏 |
| 0 | 1 | 2 | 3 | 4 | 5 | 6 |

2. Please describe the severity of the side-effects that **you think are due to the medications taken for depression during the past week**. Please circle the most serious side-effects you have experienced in the past week.

| No side effects | Minimal | Mild | Medium | Significant | Severe | Intolerable |
| --- | --- | --- | --- | --- | --- | --- |
| 🞏 | 🞏 | 🞏 | 🞏 | 🞏 | 🞏 | 🞏 |
| 0 | 1 | 2 | 3 | 4 | 5 | 6 |

3. Please describe the extent to which the **side-effects of medication due to depression** affect the function of daily life **in the past week**. Please circle the best matching degree of how it affected you in the past week.

| No effect | Minimal effect | Mild effect | Medium effect | Significant effect | Severe effect | Cannot continue with daily life |
| --- | --- | --- | --- | --- | --- | --- |
| 🞏 | 🞏 | 🞏 | 🞏 | 🞏 | 🞏 | 🞏 |
| 0 | 1 | 2 | 3 | 4 | 5 | 6 |

**Grading**

Questions 1 and 2 (frequency and severity) provide information to clinicians, but **they are not used for final scoring**

The final score is only from question 3 – the burden, the clinical significance of the score is as follows:

0-2 = Can continue the current treatment unless there is a problem with safety or symptom control

3-4 = Side effects require further treated (e.g. dose reduction)

5-6 = Change treatment plan (e.g. dose reduction or change medications)
